# The Differentiation of Rat Oligodendroglial Cells Is Highly Influenced by the Oxygen Tension: In Vitro Model Mimicking Physiologically Normoxic Conditions

**DOI:** 10.3390/ijms19020331

**Published:** 2018-01-24

**Authors:** Justyna Janowska, Malgorzata Ziemka-Nalecz, Joanna Sypecka

**Affiliations:** NeuroRepair Department, Mossakowski Medical Research Centre, Polish Academy of Sciences, 5 Pawinskiego Street, 02-106 Warsaw, Poland; jjanowska@imdik.pan.pl (J.J.); mnalecz@imdik.pan.pl (M.Z.N.)

**Keywords:** oligodendrocyte progenitor cells, myelinogenesis, hippocampal organotypic slices, cell proliferation, oligodendrocyte maturation myelin, myelin protein amounts, physiological normoxia, culture density, serum-free culture

## Abstract

Oligodendrocyte progenitor cells (OPCs) constitute one of the main populations of dividing cells in the central nervous system (CNS). Physiologically, OPCs give rise to mature, myelinating oligodendrocytes and confer trophic support to their neighboring cells within the nervous tissue. OPCs are known to be extremely sensitive to the influence of exogenous clues which might affect their crucial biological processes, like survival, proliferation, differentiation, and the ability to generate a myelin membrane. Alterations in their differentiation influencing their final potential for myelinogenesis are usually the leading cause of CNS dys- and demyelination, contributing to the development of leukodystrophic disorders. The evaluation of the mechanisms that cause oligodendrocytes to malfunction requires detailed studies based on designed in vitro models. Since OPCs readily respond to changes in local homeostasis, it is crucial to establish restricted culture conditions to eliminate the potential stimuli that might influence oligodendrocyte biology. Additionally, the in vitro settings should mimic the physiological conditions to enable the obtained results to be translated to future preclinical studies. Therefore, the aim of our study was to investigate OPC differentiation in physiological normoxia (5% O_2_) and a restricted in vitro microenvironment. To evaluate the impact of the combined microenvironmental clues derived from other components of the nervous tissue, which are also influenced by the local oxygen concentration, the process of generating OPCs was additionally analyzed in organotypic hippocampal slices. The obtained results show that OPC differentiation, although significantly slowed down, proceeded correctly through its typical stages in the physiologically relevant conditions created in vitro. The established settings were also conducive to efficient cell proliferation, exerting also a neuroprotective effect by promoting the proliferation of neurons. In conclusion, the performed studies show how oxygen tension influences OPC proliferation, differentiation, and their ability to express myelin components, and should be taken into consideration while planning preclinical studies, e.g., to examine neurotoxic compounds or to test neuroprotective strategies.

## 1. Introduction

Oligodendrocyte progenitor cells (OPCs) are one of the major cell populations within the central nervous system (CNS). They were shown to constitute about 7–9% of cells in the white matter and 2–3% in the gray matter [1,2]. After undergoing a multistage differentiation process orchestrated by a number of exogenous stimuli, they acquire the ability to myelinate axons within the CNS, assuring a fast and efficient signal propagation [3,4]. However, apart from being the precursors of the mature myelinating oligodendrocytes, OPCs, which are also recognized as neural/glial antigen 2 (NG2^+^) cells, are known to play additional roles in the nervous tissue. They confer a trophic support to their neighboring cells, stimulating neurogenesis and the differentiation of neurons by releasing neurotrophins (BDNF, NT-3), as well as exert an immunomodulatory effect in a pathophysiological environment [5,6,7]. The myelinating oligodendrocytes supply neurons with metabolic substrates, like glycogen-derived pyruvate and lactate, via the monocarboxylate transporter 1, and enwrap axons with multilayered myelin sheaths to protect them from exogenous noxious stimuli [8,9,10].

To gain the ability to generate the highly specified proteolipid myelin membrane, oligodendrocyte progenitors undergo a complex maturation process, comprising numerous molecular and morphological changes. During the subsequent steps of differentiation, a number of long and branched cell processes are elaborated, and myelin components are expressed and intracellularly transported to be incorporated into the emerging membrane. The entire process is known to be precisely guided by extracellular clues present in the local microenvironment, among many other signals derived from the neurons that are to be myelinated [11,12,13,14]. Actually, one of the most important features of OPCs is their susceptibility to the influence of local alterations in tissue homeostasis [15,16,17]. Depending on the type and intensity of the received stimuli, OPCs respond either by limiting their survival or by increasing their rate of proliferation [18,19,20]. Furthermore, it is also hypothesized that, at least in in vitro studies, the fate of OPCs could also be regulated by local soluble paracrine signals, and finally the cells can give rise to neurons [21,22]. 

Taking the above into consideration, OPCs exhibit an extreme sensitivity to the influence of exogenous signals which could trigger various cell responses, like, for instance, apoptosis, an increase in the rate of proliferation, or an arrest in the process of their differentiation. In this context, oxygen tension, which is one of the most important physiological parameters of tissue homeostasis, might have a significant impact on cell biology [23,24,25]. It has also been hypothesized that a temporal limitation in oxygen supply and a subsequent tissue reoxygenation affect OPC survival and differentiation and actually underlie leukodystrophic disorders resulting from perinatal asphyxia [26,27,28].

Contrary to the ambient oxygen level (approximately 21%), typical for a majority of studies which have been conducted to date, the physiological oxygen tension in the nervous tissue has been reported to vary between 2% and 5%, so it is several-fold lower than in standard laboratory conditions [29,30]. Keeping in mind the great number of neurological diseases with a confirmed pathology resulting from CNS hypo- or demyelination [31,32], extensive in vitro studies are to be planned and performed to estimate disease mechanisms and to test novel potential therapeutics. In this context, strictly defined and controlled culture conditions should be established for expanding and maintaining oligodendroglial cells in vitro, in an environment relevant to the physiological parameters. 

To address this issue, we performed studies on the impact of the oxygen level and cell culture density on neonatal rat progenitor cells proliferation, differentiation, and ability to express myelin components when cultured in vitro in restricted conditions. Taking into consideration that other components of the nervous tissue are also supposed to be influenced by the concentration of oxygen in the local milieu, and in this way might also contribute to modulating the investigated processes, cultures of organotypic hippocampal slices were additionally used to mimic the physiological tissue environment. 

## 2. Results 

**Figure 1:**
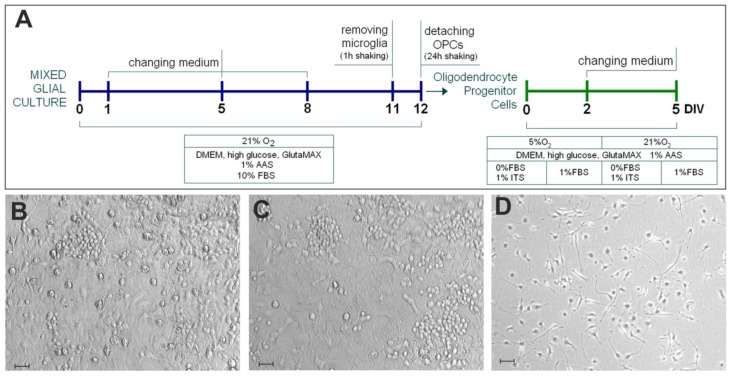
Establishing the monocultures of oligodendroglial progenitors in vitro. (**A**) A scheme of oligodendrocyte progenitor cells (OPC) isolation from the primary mixed glial culture (blue timeline) and their subsequent differentiation in four variants of cell culture conditions (green timeline); (**B**) live image of a mixed primary glial cell culture obtained from the brains of neonatal rats; (**C**) the cell culture after shaking off the microglial fraction; (**D**) OPC monoculture 24 h after cell seeding. The scale bar corresponds to 50 µm.

To examine the influence of physiological normoxia on oligodendrocyte progenitor proliferation and their differentiation into mature cells capable of expressing myelin components, the isolated OPCs (≥98% of homogeneity) were cultured either under ambient oxygen tension or in conditions mimicking the extracellular oxygen concentration (5%) present in vivo in the nervous tissue. Since oligodendrocytes are neural cells of complex morphology characterized by multibranched cell processes useful in searching the axons to be myelinated, cell culture density is also supposed to influence the process of cell differentiation. The third prerequisite of the experimental scheme was to apply strictly controlled culture conditions (by using a culture media with no added supplements) to eliminate the factors potentially influencing the processes of cell proliferation and differentiation. Therefore, the scheme of the planned investigation (Figure 1) was based on collating the effects of various culture conditions on the maturation process of oligodendroglial cells: i.e., low (1.5 × 10^4^ cells/cm^2^) versus high (5 × 10^4^ cells/cm^2^) initial culture density, the influence of ambient (21%) versus physiological (5%) oxygen concentration, and the application of serum-free versus low-serum (1% fetal bovine serum, FBS) culture media.

### 2.1. The Cell Culture Density Influences Oligodendrocyte Differentiation

The first assessed parameter tested for its effects on the growth of oligodendrocytes in vitro was cell culture density. To address this issue, cells were seeded at either low or high density, then cultured in serum-free medium under physiological normoxia (5% O_2_) for 2 days.

**Figure 2:**
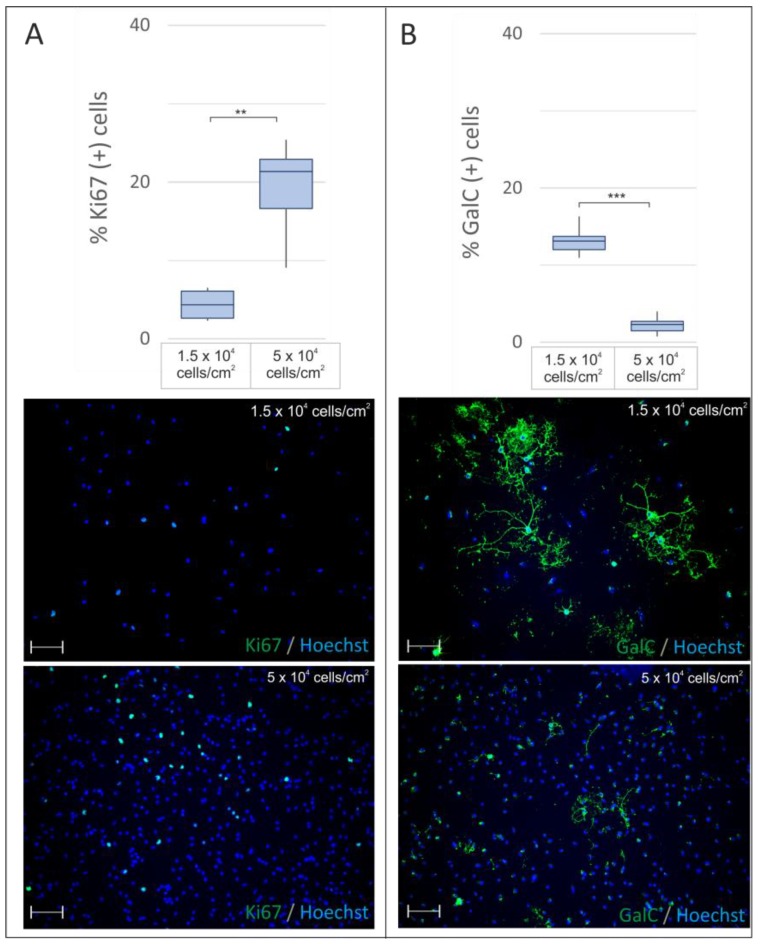
The influence of the cell seeding density on OPC proliferation (revealed by Ki67 immunostaining, **green**) and differentiation (estimated by GalC expression, **green**) determined after culturing the cells for 48 h in serum-free conditions in physiological normoxia. (**A**) Cells seeded at a high density (5 × 10^4^/cm^2^) divide approximately five-fold more frequently than those cultured in low density (1.5 × 10^4^/cm^2^), as indicated by Ki67 presence in the cell nuclei; (**B**) cell differentiation, verified by the presence of GalC^+^ oligodendrocytes, is highly influenced by the cell culture density. When cultured in low density, GalC^+^ cells are significantly more numerous and they are characterized by a much more complex, branched morphology. The cell nuclei were labelled with Hoechst 33258 (**blue**). The scale bar is the equivalent to 100 µm. The calculated differences were considered statistically significant when ** *p* < 0.05; *** *p* < 0.001.

The proliferating cells were identified by the presence of Ki67 protein in their nuclei, while the progress in the differentiation process was verified by visualizing GalC in the cell membrane. The results of the immunocytochemical analysis showed that if OPCs were plated sparsely, they divided significantly (*p* = 0.0001) less frequently than in high cell density (4.37 ± 1.07% versus 19.25 ± 1.54% of the total cell fraction) (Figure 2A). However, the availability of space among sparsely plated cells turned out to be much more permissive for cell maturation, resulting in a significantly (*p* = 0.0001) increased number of GalC-positive cells (median 13.17 ± 0.76%) compared with cells cultured in high density (median 2.17 ± 0.38%) (Figure 2B). Moreover, cell morphology in low-density cultures was characterized by more complex, ramified processes (Figure 2B). 

### 2.2. Normoxic Conditions Promote Cell Proliferation and Support the Abundancy of the Progenitor Fraction in In Vitro Oligodendroglial Primary Monocultures

**Figure 3:**
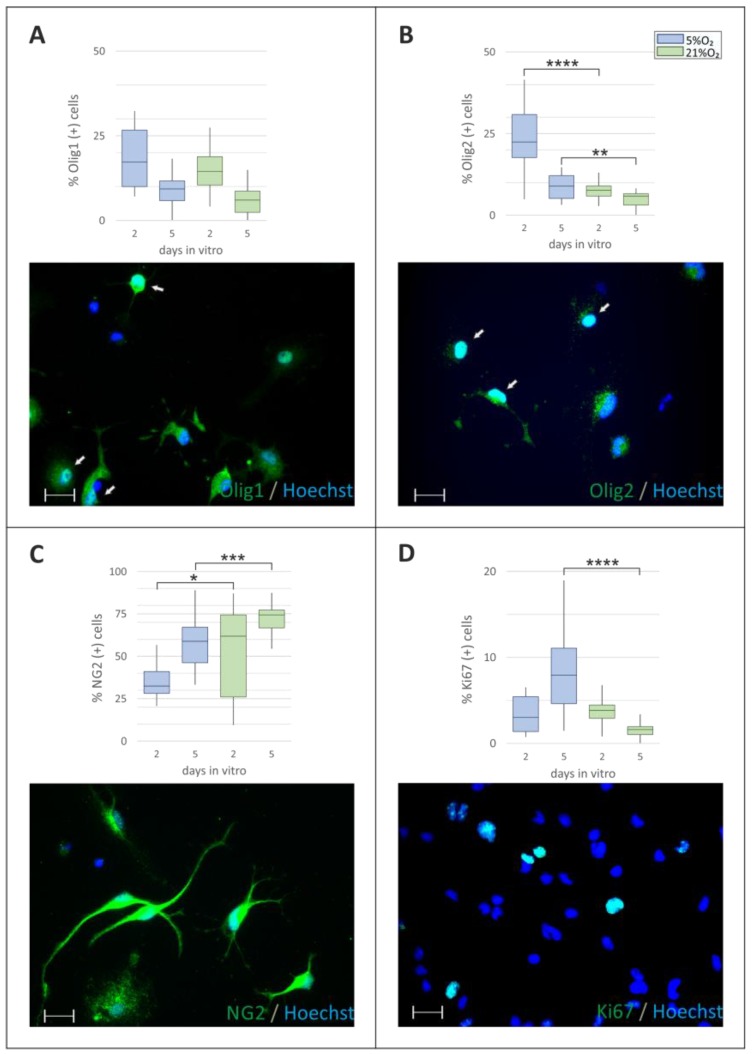
Quantification of immunolabeled oligodendrocyte progenitors in different cell culture conditions. Cell cultures were fixed either after 2 or 5 days in vitro. Blue bars show cells maintained in physiological normoxia (5% O_2_) and green bars represent cells kept at the standard oxygen level (21% O_2_). (**A**) Expression of the lineage-specific transcription factor Olig1 (**green**), which is detected in the cell cytoplasm on the 2nd DIV (white arrows) and gradually decreases during cell maturation; (**B**) Expression of the transcription factor Olig2 (**green**), which is relevant for the early stages of oligodendrocyte differentiation (white arrows); (**C**) Progenitor cells recognized by the presence on their surface of the NG2 marker; (**D**) Proliferating cells characterized by the presence of the Ki67 marker in the cell nuclei. The cell nuclei were visualized with Hoechst 33258 solution (**blue**). The scale bar is equivalent to 20 µm. The calculated differences were marked as the significant if: * *p* < 0.05, ** *p* < 0.01; *** *p* < 0.001, **** *p* < 0.0001.

After determining the optimal cell culture density, oligodendrocyte differentiation in distinct oxygen conditions was analyzed by immunostaining with a panel of developmental stage-specific antibodies. Firstly, the total number of oligodendroglial progenitors, recognized by their characteristic markers, namely, by the presence of chondroitin sulfate proteoglycan (NG2) in the cell membrane and by the expression of the lineage-specific transcription factor Olig1, was assessed. As indicated by the immunocytochemical analysis, the number of progenitors in a cell culture strongly depends on both the oxygen tension and the trophic support provided by a very low concentration of serum. Since oligodendrocyte differentiation from progenitor cells proceeds relatively quickly in vitro, the abundancy of the progenitor fraction was examined on both the 2nd and the 5th day in vitro (DIV). The obtained data indicated that the expression of the lineage-specific transcription factors Olig-1 (Figure 3A) and Olig-2 (Figure 3B) was highly dependent on the oxygen level and was significantly upregulated under normoxic conditions at both the analyzed time points. Conversely, the number of cells expressing NG2, which is an integral component of the cell membrane, increased during cell culturing in ambient oxygen concentration (34.42 ± 2.6% versus 51.17 ± 8.43% on the 2nd DIV and 57.81 ± 2.9 versus 72.95 ± 1.87% on 5th DIV) which could indicate an acceleration in cell differentiation (Figure 3C). Normoxic conditions were also shown to exert a considerable effect on the rate of cell proliferation (8.33 ± 1.14% in 5% O_2_ versus 1.57 ± 0.19% in 21% O_2_, on the 5th DIV in serum-free medium, *p* < 0.0001) as indicated by the expression of Ki67 protein in the nuclei of the dividing cells (Figure 3D).

### 2.3. The Ambient Oxygen Tension Accelerates the Maturation of Oligodendrocytes 

**Figure 4:**
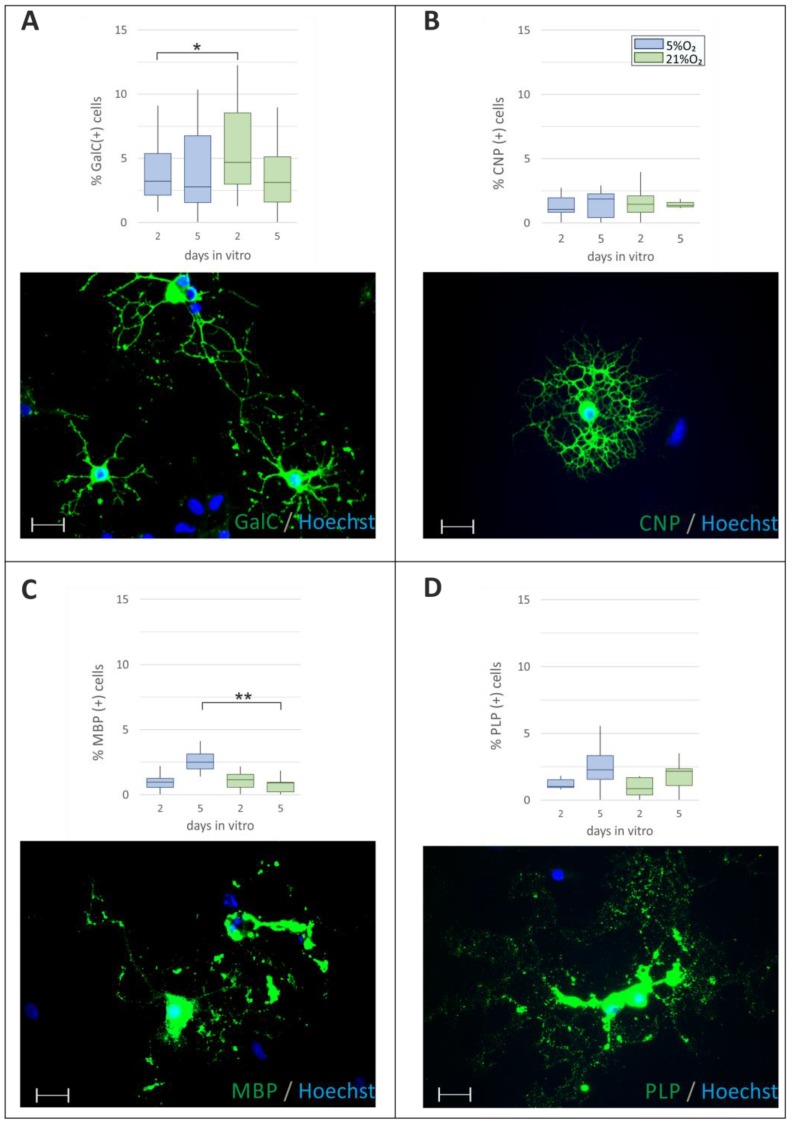
Quantification of maturating oligodendrocytes maintained in different culture conditions for either 2 or 5 DIV. The cells were cultured either in 5% (**blue bars**) or 21% O_2_ (**green bars)**. (**A**) Differentiating, multiprocessed cells are characterized by the presence of the GalC antigen on the cell surface; (**B**) Expression of CNP (2′,3′-Cyclic-nucleotide 3′-phosphodiesterase), the characteristic marker of the oligodendroglial lineage; (**C**) Detection of myelin basic proteins (MPB) in differentiated oligodendrocytes with branched cell processes; (**D**) Presence of proteolipid protein (PLP) in mature cells. The cell nuclei were labelled with Hoechst 33258 (**blue**). The scale bar corresponds to 20 µm. The calculated differences were considered statistically significant if: * *p* < 0.05, ** *p* < 0.01.

After examining a number of progenitors and measuring their proliferation rate in different cell culture conditions, the progress in differentiation was evaluated by staining the cells with an antibody directed against galactocerebroside (GalC), a protein characteristic of cells elaborating a net of cell processes. However, for this membrane component found in cells during the middle stage of their maturation, a significant (*p* = 0.02) influence of the oxygen level was noted on the 2nd DIV in serum-free media (3.82 ± 0.38% of GalC^+^ cells in 5% O_2_ versus 5.38 ± 0.53% in 21% O_2_) (Figure 4A). Therefore, the next marker analyzed for its expression at the two time points under distinct oxygen conditions was the enzyme 2′,3′-cycling nucleotide 3′-phosphodiesterase (CNPase). Although the anti-CNP staining allowed the visualization of well-elaborated and highly ramified cell processes, which indicated advanced cell differentiation, the number of cells in all the investigated experimental groups was approximately similar (Figure 4B). The only increase (approximately 3-fold) in the number of CNP^+^ cells was observed when a low serum concentration was present in the culture media. 

The most relevant indicator of cell maturation is, however, the presence of myelin components. Their expression confirms that an oligodendrocyte is capable of myelinogenesis, which is the end-point of the maturation process. Therefore, antibodies against the most typical myelin-associated proteins were applied to estimate the progress in cell differentiation. While myelin basic proteins (MBP) are embedded in the myelin layers, the proteolipid protein (PLP) is the major myelin constituent, characterized by four transmembrane domains. The expression of MBP under normoxic conditions varied significantly compared to that observed under ambient oxygen tension (*p* = 0.0009 and *p* = 0.0029, respectively) (Figure 4C), which was most visible on the 5th DIV in both the 1% serum-containing media and the serum-free conditions. Such significance was not observed in the case of PLP detection (Figure 4D). 

**Figure 5:**
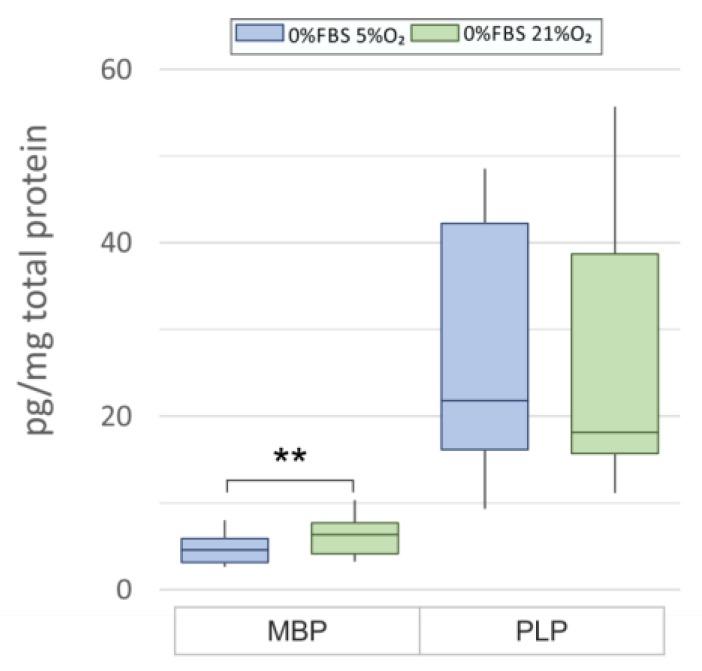
Quantitative analysis of the major myelin components in oligodendrocytes cultured in distinct oxygen concentrations (physiologically relevant 5% O_2_ versus standard 21% O_2_) on the 5th DIV. Statistical significance is ** *p* < 0.01.

Taking into consideration that the ability for myelinogenesis is a major determinant of cell maturity, the results obtained by the immunocytochemical methods were verified biochemically by performing quantitative measurements by ELISA tests. Accordingly, the amount of MBP in the oligodendroglia on the 5th DIV varied between approximately 4.707 ± 0.47 pg/mg of total proteins under normoxic conditions and 6.28 ± 0.61 pg/mg of total proteins under ambient conditions (*p* = 0.001) (Figure 5). The content of PLP, which is an abundant compound of mature oligodendroglia, was higher and its quantities were estimated as 28.34 ± 3.97 pg/mg of total proteins in physiological oxygen concentration and 26.23 ± 4.74 pg/mg of total proteins when the cells were cultured at an oxygen level typical of the surrounding environment (21%). 

**Figure 6:**
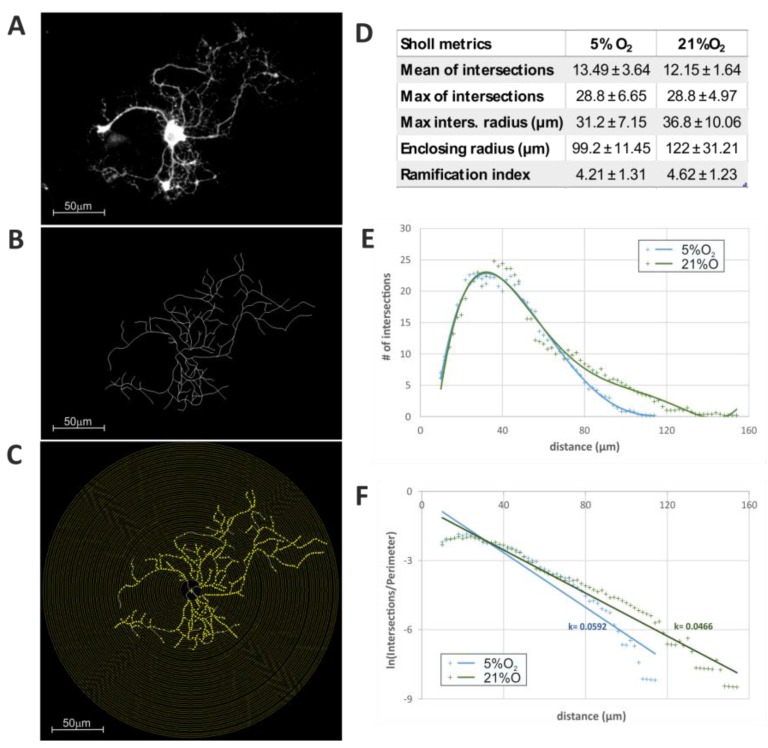
Evaluation of the influence of environmental oxygen concentration on oligodendrocyte (CNP^+^ cells) differentiation, based on Sholl analysis of cell morphology on the 2nd DIV. The comparison of the length and complexity of the elaborated cell projections indicates that physiological normoxia (5% O_2_) is permissive for the elongation of oligodendroglial processes, which is necessary for searching axons to be myelinated. (**A**) Original, 8-bit image of the cell; (**B**) Black and white masks of cells prepared by means of the NeuronJ software. A series of concentric circles around the cell bodies allowing for marking the intersections by application of the Sholl plugin to ImageJ is shown; (**C**) Concentric circles around the cell bodies drawn with Sholl plugin to Fiji; the yellow dots mark the intersections of the cell process with consecutive circles; (**D**) Sholl metrics based on samples’ data; (**E**) The linear profile presents the average number of intersections versus the distance from the cell body; (**F**) The semilog plot shows the average number of intersections normalized to the perimeter of the circles. The slope of the linear regression (Sholl’s Regression Coefficient—k) is the measure of the change in density of the branches with respect to their distance from the center.

Taken together, these results suggest that the cells were still actively maturating on the 5th DIV. Moreover, when taking a close look at the cell morphology visualized by immunostaining with a panel of various cell markers, a variation in the complexity of the cell processes was noticed. To verify if there was any significant diversity, the length and ramification of extended processes were measured by means of the Sholl analysis (Figure 6A,B). Accordingly, the means of intersections (12.14–13.5 µm) and maximum of intersections (28.8 µm) were similar for both culture conditions, indicating an advanced process branching, resulting in a complex cell morphology (Figure 6C,D). 

However, the analysis of additional advanced quantitative descriptors (like the radius of the highest count of intersections, enclosing the radius and the Sholl regression coefficient) pointed to compelling differences in the length of the elaborated cell arbors. Namely, the cells cultured under standard oxygen conditions were characterized by longer processes (122 ± 31.2 µm) in comparison to the cells grown in physiological normoxia (99.2 ± 11.45 µm). The performed morphometric analysis supported the observation that standard oxygen conditions accelerated cell maturation, which could be noticed even during a relatively short, 5-day long in vitro culture.

### 2.4. The Supplementation with a Low Serum Concentration Significantly Affects Oligodendroglial Differentiation 

While the most significant differences between culture conditions were observed for the initial markers of OPC maturation (Olig1, Olig2, NG2), the later markers stained cells with well-elaborated cell processes, as well as those whose morphology changed just before they detached and underwent cell death (for instance, in Figure 4C, the MBP^+^ cell in the right upper corner). The addition of even low (1%) amounts of serum enhanced the rate of cell proliferation (Table 1), as well as their differentiation (based on the number of MBP^+^ cells) in both oxygen conditions and time points (Figure 4C). However, taken together, the results show that oligodendrocytes proliferate, differentiate, and could be cultured in vitro for several days in restricted, serum-free conditions. 

### 2.5. Cell Proliferation and Differentiation under Normoxic Conditions in Organotypic Hippocampal Slices

The in vitro differentiation of cells in monocultures has many advances, like, for instance, the possibility to test the influence of various parameters on cell biology, or the possibility to precisely visualize and subsequent analyze all cell extensions. These advances were very useful for the planned experiments and allowed to answer the main question concerning the impact of oxygen conditions on oligodendrocyte maturation. In the nervous tissue, however, the cells are interdependent, and, in particular, the signals derived from neurons are known to regulate oligodendrocyte maturation. Moreover, taking into account the obtained results concerning the dependence of oligodendrocyte differentiation on oxygen concentration, a similar effect could be hypothesized for neuronal physiology. 

**Figure 7:**
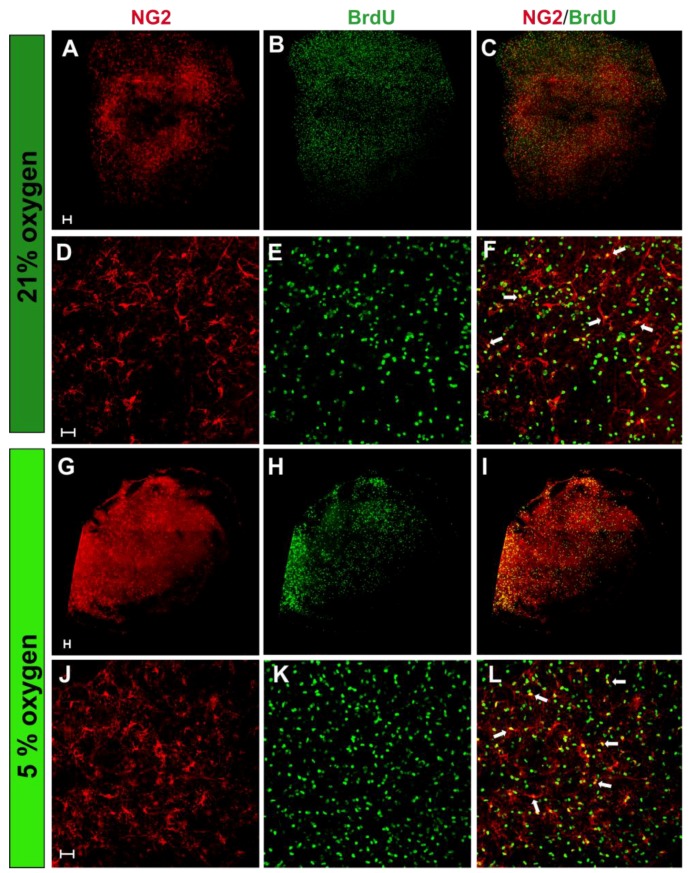
Hippocampal organotypic slices cultured either in standard (**A**–**F**) or physiologically normoxic (**G**–**L**) conditions. Oligodendroglial progenitors were stained with anti-NG2 antibody (**red**), while proliferating cells were visualized by BrdU (5-bromo-2′-deoxyuridine) incorporation (**green**). Double-labelled cells (white arrows) indicate the newly generated oligodendrocytes in the slices during a 7 day long culture in serum-free medium. The scale bar is equivalent of 50 µm.

**Figure 8:**
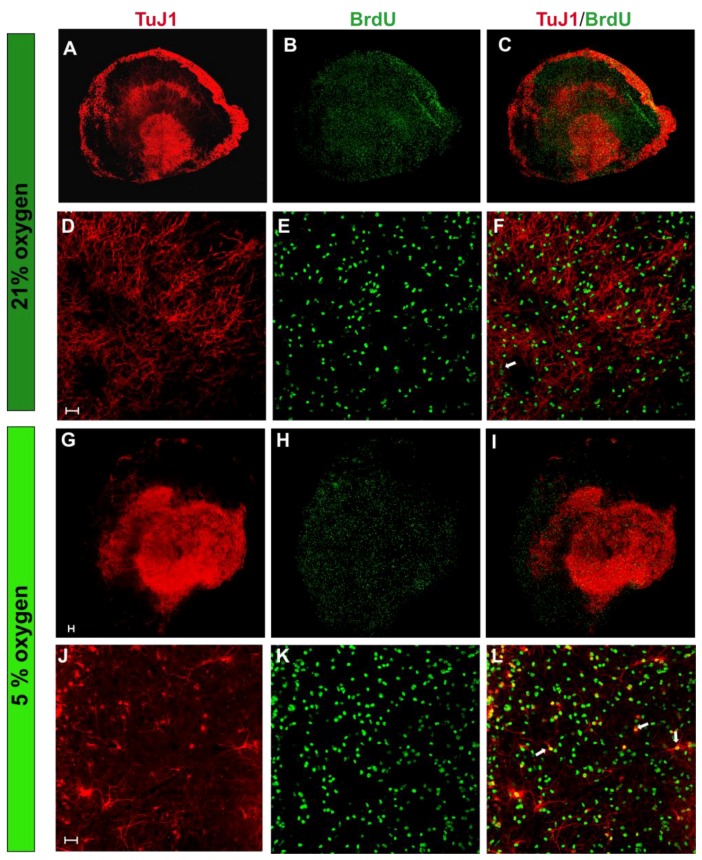
Proliferating neurons within hippocampal slices cultured for 7 DIV, visualized by TuJ1 antibody (**red**) and BrdU (**green**) in the cell nuclei of the dividing cells. (**A**–**C**) The tile scan of an entire hippocampal slice cultured in standard oxygen conditions (21% O_2_); (**D**–**F**) Magnification of the hippocampal slice, allowing to distinguish the double-labelled (TuJ1^+^/BrdU^+^) dividing cells (**white arrows**); (**G**–**I**) Tile scan of an hippocampal slice cultured in physiologically normoxic conditions (5% O_2_); (**J**–**L**) Enlargement of the slice, showing the colocalization of the neuronal marker (Tuj1) and BrdU, staining the newly derived cells (whithe arrows). The scale bar is equivalent of 50 µm.

To address this issue, hippocampal organotypic slices were used to assess oligodendrocyte differentiation in ex vivo nervous tissue cultures under normoxic conditions. The hippocampus, distinguished by its characteristic neurogenic regions, is an area of intense neuronal derivation and maturation. Culturing hippocampal slices ex vivo enables the preservation of the tissue architecture and integrity. To estimate the number of oligodendroglial progenitors and their proliferation under both tested oxygen concentration, a double-labeling of cells was performed after 7 days in serum-free media (Figure 7A–D). The newly generated cells were identified by BrdU incorporation into the newly synthesized DNA of the dividing cells. A statistical analysis indicated a significantly increased number of oligodendroglial progenitors in cultures at the physiological oxygen concentration (153.2 ± 6.97 of NG2^+^ cells per 606.3 µm × 606.3 µm area) compared to cultures at the ambient oxygen concentration (118.5 ± 7, significant at *p* = 0.0019). Furthermore, the fraction of proliferating progenitors (NG2^+^/BrdU^+^) was increased by 30% under normoxic condition (*p* = 0.0014). Similarly, when the generation of neurons in the hippocampal slices was estimated by visualizing the colocalization of Tuj1 marker and BrdU, it turned out that normoxic conditions strongly supported neuronal proliferation (Figure 8). While, in standard conditions, approximately 4.31 ± 0.8 cells were dividing, their number increased by as much as 250% in a corresponding area in normoxic conditions (*p* < 0.0001). 

**Figure 9:**
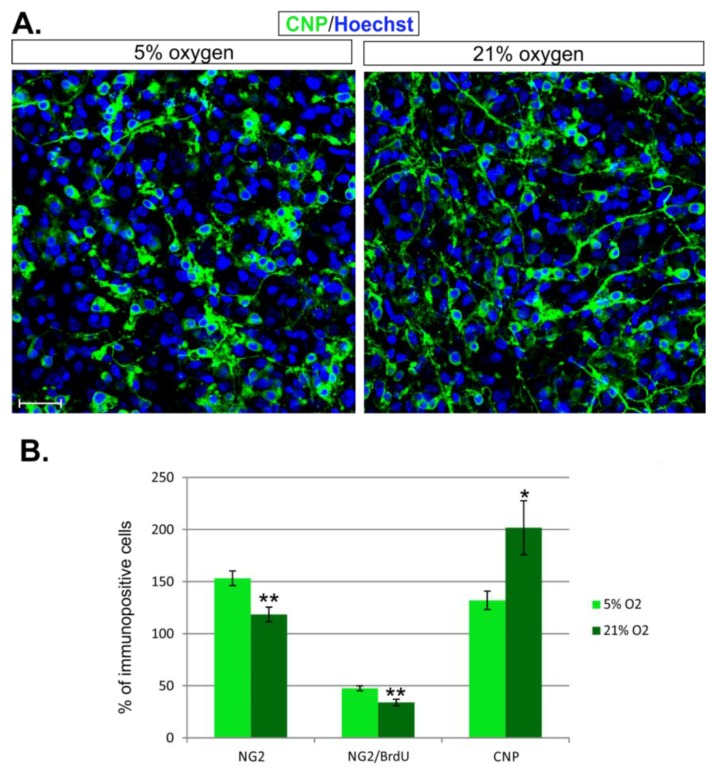
The influence of distinct oxygen conditions on oligodendrocyte proliferation and differentiation. (**A**) The CNP-positive oligodendrocytes (**green**) visible in hippocampal slices after 7 DIV; The cell nuclei were labelled with Hoechst 33258 (**blue**); (**B**) The diagram shows the total number of OPCs, dividing progenitors, and differentiating oligodendrocytes in hippocampal slices cultured in distinct oxygen concentrations. The scale bar is equivalent to 20 µm. The calculated differences were regarded as statistically significant if * *p* < 0.05, ** *p* < 0.01.

The evaluation of the total content of differentiating oligodendrocytes in the hippocampal slices after 7 days in distinct oxygen concentrations also revealed significant differences. While CNP^+^ cells accounted for approximately 132 ± 21.65 per 606.3 µm × 606.3 µm area in physiological normoxia, their amount was elevated to 202.7 ± 63.51 in standard conditions (*p* = 0.0274) (Figure 9A,B). In summary, the data coming from the immunohistochemical examination indicate that the normoxic conditions support the abundancy and proliferation of the progenitor fraction, while the standard conditions accelerate oligodendrocyte differentiation (Figure 9B). 

## 3. Discussion

To acquire the ability to express myelin components and to form the highly specialized myelin membrane which is the end-point of oligodendrocyte maturation, precursor cells undergo a precise and multistage differentiation process. In vivo, this process is known to be instructed by a plethora of extracellular signals present in the local microenvironment. Among many others, the instructive clues include the presence of extracellular matrix components (laminin, fibronectin) affecting the formation of cell extensions and their elongation [33], hormones (like triiodothyronine, T3) promoting cell maturation [34,35,36], and trophic factors released by neighboring neural cells Platelet-Derived Growth Factor-AA (PDGF-AA, Fibroblast Growth Factor 2 (FGF2), Leukemia Inhibitory Factor (LIF), Ciliary neurotrophic factor (CNTF). The process of myelinogenesis is also enhanced by the electric activity of neurons providing signals about targets to be enwrapped with myelin sheaths [37,38,39]. Interestingly, although strongly influenced by the environmental clues, oligodendrocytes are able to complete their differentiation process both in vitro and in vivo in the absence of neurons to be myelinated [40,41]. This ability of oligodendrocytes to terminally maturate even when maintained as monocultures, enables their use as in vitro models for various applications, like, for instance, testing drugs and neurotoxicants, or disease modelling [42,43,44,45].

However, to consider them in preclinical investigations, the planned studies have to be performed in conditions maximally resembling the physiological environment. The discrepancies between laboratory and in vivo conditions aroused many controversies [46]. One of them concerns culturing cells and tissue slices in atmospheric oxygen tension (close to 21%), while physiological normoxia (physioxia) is known to range between 2–9% O_2_ [47]. 

Fluctuations of the oxygen level are known to influence in situ crucial cell functions by their impact on microRNAs (miRNAs), which are short (19–24 nucleotides) RNA molecules regulating gene expression by binding to the 3′-untranslated region (3′-UTR) of mRNA targets [48,49]. Another intracellular signaling pathway depending on the local oxygen concentration operates via hypoxia inducible factors (HIFs) and is known to regulate cell survival and differentiation [50,51]. In the case of oligodendrocytes, activated HIFs promote angiogenesis through Wnt7 signaling pathway and arrest cell differentiation unless the extracellular O_2_ level increases and deactivates HIF1α and HIF2α by specific oxygen-dependent enzymes (asparaginyl and prolyl hydroxylases) [52,53]. When the microvasculature is well developed and the trophic support from the circulating blood is provided, the energy-consuming process of oligodendrocyte differentiation proceeds. Disturbances in oxygen supply to the nervous tissue, especially during perinatal asphyxia, are thought to contribute to the inhibition of oligodendrocyte differentiation and subsequently to the inefficient or aberrant myelination of the brain, typical of leukodystrophic disorders. 

Our studies based on cultivating neonatal rat oligodendrocytes in a physiologically relevant oxygen tension revealed the influence of the oxygen concentration on cell proliferation and maturation. To evaluate the progress in cell differentiation, we analyzed the cell markers attributed to the sequential stages of oligodendrocyte differentiation [54]. Accordingly, the oligodendroglial commitment of the neural stem cells could be verified by the expression of the lineage-specific basic helix-loop-helix (bHLH) transcription factors Olig1 and Olig2 [55,56,57,58]. The progenitor cells were identified by the presence on their surface the chondroitin sulfate proteoglycan (also known as neural/glial antigen 2, NG2), which is a classic OPC marker. Interestingly, the progenitors are characterized by a different morphology: from small and bipolar to multibranched cells with relatively long cellular extensions. Because of their complex appearance, they have been named *polydendrocytes* [59,60]. However, even the cells with fine developed cell processes are considered undifferentiated oligodendrocytes, since they do not initiate myelinogenesis, but remain scattered in both the white and the grey matter without proceeding to the next stage of oligodendroglial differentiation. 

An advance in the maturation process is usually recognized by the expression of the GalC marker, attributed to immature oligodendrocytes. The end-point of the differentiation process was determined on the basis of both the cell morphology and the expression of major myelin constituents, i.e., proteolipid protein (PLP) and myelin basic protein, which account for approximately 8% and 1% of total myelin proteins, respectively. Although both components are expressed during oligodendrocyte maturation, their intracellular trafficking is different. Namely, PLP is synthesized and transported towards the emerging myelin membrane in recycling endosomes and lysosomes through transcytosis pathway [61,62,63]. Conversely, the mRNA coding for MBP binds to ribonucleoprotein A2 (hnRNP A2) and is then transported in granules containing components of the translation machinery even to the distal ends of the oligodendroglial processes, where the myelin membrane is formed. In this way, the translation of MBP mRNA occurs in situ during myelin biogenesis [64,65]. These two proteins are known to stabilize myelin by zippering the apposed layers and contributing to its multilamellar structure [66,67,68,69]. 

The in vitro recapitulation of the physiological functions of oligodendrocytes includes the evaluation of cell proliferation. OPCs are known to be one of the major populations of cycling cells within the CNS, readily responding to microenvironmental clues (including pathological signals triggered by insults or neuroinflammation) by increasing their proliferation rate [2]. Therefore, the preserved proliferation ability of the cells dividing in vitro confirms that OPCs functions are maintained during cell culturing. The presented study shows that this cell feature is also highly influenced by the oxygen concentration in the milieu. Namely, the cell proliferation rate was nearly 2 times higher (for oligodendroglia) or even 5 times higher (in case of neurons) in physiological normoxia than in atmospheric oxygen concentration. This observation is in line with a previous study reporting that the normoxic oxygen concentration is permissive for sustaining the progenitor fraction [70] and with another study describing the propagation of human neural progenitors in vitro and the subsequent generation of the oligodendroglial lineage [71]. Accordingly, the standard oxygen tension was shown to accelerate the process of cell maturation, resulting in a high yield of GalC^+^ oligodendrocytes. This disclosed dependence is important especially when studying in vitro cell responses to specific conditions (selected biological factors, applied drugs, pathological signals). The data indicate also that both the cell cycling and their differentiation are significantly influenced by the addition of serum. Usually, the supplementation of culture media with low serum doses is beneficial for maintaining primary cultures for a prolonged period of time. The undefined serum composition precludes, however, any preclinical studies and, additionally, modifies cell biology. In the present study, even 1% of FBS present in the milieu significantly promoted cell differentiation. 

The in vitro generation of cells with myelinating potential is not only dictated by physical parameters present in the cell incubator (usually enriched with 5% CO_2_ and 95% humidity), by also by the seeding density of the cells. Contact inhibition of growing cells should be always considered, especially in the case of cells with long or branched extensions. 

In conclusion, the presented study describes the proliferation of oligodendrocyte progenitors and their differentiation in optimal culture conditions, relevant in many aspects to the physiological parameters in which the cells are functioning. This protocol enables broad applications of in vitro primary oligodendroglial monocultures for both basic and preclinical studies. Physiological normoxia was shown to exert a neuroprotective effect in vitro and ex vivo, in comparison to standard culture conditions in terms of promoting neural cell proliferation and supporting the abundancy of progenitors, which could serve as a cell reservoir for neurorestorative processes. 

## 4. Materials and Methods

### 4.1. Primary Culture of Rat Oligodendrocyte Progenitors In Vitro

Mixed glial cultures were established from 2-day-old Wistar rats (*n* = 18) bred in the Animal Care Facility of the Mossakowski Medical Research Centre. The detailed procedure was described previously [22]. The protocol was approved by the Local Ethics Committee on Animal Care and Use. Briefly, cerebral hemispheres were mechanically dispersed in Dulbecco’s Modified Eagle’s Medium high-glucose (Gibco, Gaithersburg, MD, USA) supplemented with 10% fetal bovine serum (FBS, Gibco, Gaithersburg, MD, USA) and 1% Antibiotic-Antimycotic Solution (Sigma, Mendota Heights, MN, USA), using a Pasteur pipette and a 1.2 mm Luer-Lock needle. Cells were then filtered through a Hydrophilic Nylon Net Filter (Millipore, Burlington, MA, USA) with 41.0 µm pore size and plated onto 75 cm^2^ culture flasks (NUNC, Roskilde, Denmark) coated with 0.1 mg/mL poly-l-lysine (Sigma) (Figure 1A,B). The medium was changed every 2–3 days, and after 11–13 days the cells were ready to be shaken in a horizontal orbital shaker (Stuart) to obtain the oligodendrocyte progenitor cells. First, the flasks were preshaken for 1 h at 180 rpm to remove microglia, and, after replacing the medium, OPCs were detached during additional 18–20 h of shaking (180 rpm) (Figure 1C). After the collected suspension was spun down (1500× *g*, 10 min) and filtered (41.0 µm pore size filter, Millipore), OPCs were seeded onto 24-well plates containing poly-l-lysine-coated glass coverslips (Figure 1D) for immunocytochemistry and onto 6-well plates coated with poly-l-lysine for further quantitative biochemical measurements. To evaluate the influence of the culture density on cell proliferation and maturation, OPCs were plated either at a low (1.5 × 10^4^ cells/cm^2^) or high (5 × 10^4^ cells/cm^2^) density. The cells were grown in medium supplemented with either 1% FBS or 1% the Insulin-Transferrin-Selenium-A Solution (ITS) (Invitrogen, Carlsbad, CA, USA) and either under 5% (physiological normoxia) or 21% (standard laboratory conditions) O_2_, as presented on the scheme of the experimental design (Figure 1A).

### 4.2. In Vitro Culture of Organotypic Hippocampal Slices (OHC)

The culture of hippocampal organotypic slices was established on the basis of a protocol described in detail elsewhere [6]. Briefly, 7-day-old Wistar rats (*n* = 12) were used for brain hemispheres isolation and subsequent hippocampal slice preparation, according to the procedure approved by the IV Local Ethics Committee on Animal Care and Use (affiliated to the Ministry of Science and Higher Education). At the very beginning of the procedure, a deep hypothermia was applied to the experimental animals. The extracted hippocampi of both hemispheres, chilled on ice, were cut into 400 μm-thick slices by a McIlwain apparatus. Placed on Millicell-CM (Millipore) membranes, the slices were cultured initially in Dulbecco’s Modified Eagle Medium (DMEM) medium (Gibco) containing horse serum (25%), HBSS (25%), glucose (2 mmol/L), HEPES (5 mg/mL), B27 supplement, and an antibacterial-antimycotic solution. Starting from the second day in vitro (DIV), the serum content was gradually lowered, and, finally, from the 5th DIV onwards, the slices were cultured for the following seven days in serum-free conditions (DMEM supplemented with ITS and antibiotic solution) and in normoxic oxygen conditions (5% oxygen). To assess cell proliferation, a 5 μM solution of 5-bromo-2′-deoxyuridine (BrdU, Sigma) was added for 24 h to the slice cultures. To evaluate the incorporation of BrdU into the newly synthesized DNA of the replicating cells, on the 7th DIV of culture in serum-free and normoxic conditions, the hippocampal slices were fixed with 4% paraformaldehyde (PFA), then gently washed 3 times with an extensive amount of PBS, and finally incubated with 95% methanol for 10 min. After three additional washes with PBS, the cells were permeabilized with 2 N HCl for 10 min. In the next step, the acid was neutralized with 0.1 M sodium borate for 5 min (RT). After selecting slices with a well-preserved, characteristic tissue cytoarchitecture, immunolabeling with a set of specific antibodies recognizing subsequent stages of oligodendrocyte maturation was performed

### 4.3. Immunofluorescent Staining of Differentiating Oligodendrocytes 

Oligodendrocytes cultured for either 2 or 5 DIV were washed with PBS for 5 min, then fixed with 4% paraformaldehyde in PBS for 20 min and rinsed three times for 5 min with PBS. The unspecific binding of antibodies was prevented by incubating the fixed cells with 10% normal goat serum (Sigma) in PBS containing 0.1% Triton X-100 (Serva, Heidelberg, Germany) for 1 h at room temperature. Selected primary antibodies, diluted in PBS supplemented with 5% normal goat serum, were applied overnight at 4 °C. The list of primary antibodies used for the immunolabeling procedure included those recognizing the proliferating cells, i.e., anti-Ki67 protein (Novocastra-Leica Biosystems, Nussloch, Germany, 1:100) and anti-BrdU (Santa Cruz Biotechnology, Dallas, TX, USA, 1:100), as well as those recognizing specific markers of oligodendrocyte maturation, i.e., NG2 (Chemicon, Tokyo, Japan, 1:100), Olig1 (Merck, Kenilworth, NJ, USA, 1:1000), Olig2 (Merck, 1:500), CNP (Chemicon, 1:100), GalC (Chemicon, 1:200, Triton not used), MBP (Merck, 1:100), and PLP (Chemicon, 1:200). To visualize neurons within hippocampal slices, the TuJ1 antibody (recognizing β-tubulin III, 1:100, Sigma) was used. After washing the immunostained cells in primary cultures or hippocampal slices with PBS (3 × 5 min), the appropriate FITC-conjugated secondary antibodies (AlexaFluor, Invitrogen) were applied for 1 h RT in the dark. All the secondary antibodies used were either diluted 1:1000 for the cells or 1:500 for the slices in PBS containing 5% normal goat serum. Then, the cells and the slices were washed three times with PBS and incubated with 5 µM Hoechst 33258 (Sigma) for 15 min to visualize the cell nuclei. After covering with the Fluoromount™ reagent (Sigma), the slides were subjected to a detailed analysis using the LSM 780/ELYRA PS.1 superresolution confocal system (Carl Zeiss, Jena, Germany).

### 4.4. Biochemical Analysis of Myelin Protein Expression in Differentiating Oligodendrocytes

Cell lysates for the quantification of the selected proteins were obtained by applying the CellLytic solution (Sigma) containing a Protease Inhibitor Cocktail (Sigma, 1:100). Briefly, on the 5th DIV, oligodendrocytes cultured in serum-free medium either in 5% or 21% O_2_ were washed with PBS, and 0.4 mL of extraction reagent was added per well. The plates were incubated for 15 min on a shaker, and the lysates were then scrapped from the plates and centrifuged for 15 min, at 14,000× *g*. Total protein concentrations in the lysates were determined by DC Protein Assay (Bio-Rad, Hercules, CA, USA) according to the manufacturer’s manual. To quantify the amounts of major myelin proteins, the Sandwich ELISA kit for measuring either PLP or MBP (Abbexa, Cambridge, UK) was used according to the manufacturer’s instructions. The plates were inserted in the spectrophotometric plate reader Fluorostar Omega (BMG LabTech, Ortenberg, Germany), and the intensity of the colorimetric reaction was measured at 450 nm wave length.

### 4.5. Estimation of the Differentiating Cell Morphology 

The complexity of the elaborated oligodendroglial processes was estimated by means of the ImageJ software allowing for Sholl analysis (Sholl Analysis vs. 3.7.0, available online: https://imagej.net/Sholl) [72,73]. The most representative CNP-stained cells cultured either under 5% or 21% oxygen were chosen for a subsequent examination. The size, length, and radius of the cells were assessed by converting the typical fluorescent picture into binary 8-bit images by means of the NeuronJ software (Available online: https://imagej.net/NeuronJ). A number of quantitative descriptors was recorded, including the maximum and mean of intersections. The measurements were performed on the basis of the following parameters: starting radius, 10 µm and radius step size 2 µm. The slope of the linear regression (Sholl’s Regression Coefficient—k) is the measure of the change in density of the branches with the distance from the center.

### 4.6. Statistical Analysis

The immunofluorescently labeled cells were counted on randomly selected 5–10 visual fields on each of at least five slides from each of the three experiments. A statistical analysis of the obtained results was performed with the use of the GraphPad PRISM 5.0 La Jolla, CA, USA software. For comparing all the examined experimental variants (i.e., 0% FBS/5% O_2_, 0% FBS/21% O_2_, 1% FBS/5% O_2_, 1% FBS/21% O_2_) an one-way analysis of variance (ANOVA) followed by the Bonferroni’s Multiple Comparison Test was done. A statistical comparison of two groups was made by application of Mann–Whitney test (two-tailed with Gaussian approximation). The collected data from cell counting in in vitro cultures and their statistical analysis are presented as box and whisker charts, where the bottom and top of the box correspond to the first and the third quartile, the band inside the box is the median, while the ends of the whiskers represent the minimal and the maximal values, respectively. All data were expressed as mean ± SEM. The calculated differences were marked as significant if: * *p* < 0.05, ** *p* < 0.01; *** *p* < 0.001.

## Figures and Tables

**Table 1:** Impact of the culture conditions on OPCs proliferation and differentiation into mature cells capable of myelinogenesis.

Impact of Different Culture Conditions on OPCs	Proliferation	Maturation
OPCs and OHC in serum-free medium		
5% O_2_	↑	↓
21% O_2_	↓	↑
OPCs in medium supplemented with 1% FBS		
5% O_2_	−	↑
21% O_2_	↑	↑
OPCs in serum-free medium, 5% O_2_		
high density of culture	↑	↓
low density of culture	↓	↑

Abbreviations—OPCs: oligodendrocyte progenitor cells; OHC: organotypic hippocampal slice culture; FBS: fetal bovine serum; ↑: upregulation of the process; ↓: downregulation of the process; −: no significant changes observed.

## Abbreviations

BrdU	5-Bromo-2′-Deoxyuridine
CNP	2′,3′-Cyclic-nucleotide 3′-phosphodiesterase
DIV	days in vitro
FBS	fetal bovine serum
HIFs	hypoxia inducible factors
MBP	myelin basic proteins
NG2	chondroitin sulfate proteoglycan
OPC	oligodendrocyte progenitor cell
PLP	Myelin proteolipid protein
